# Development of the mammalian cortical hem and its derivatives: the choroid plexus, Cajal–Retzius cells and hippocampus

**DOI:** 10.1098/rsob.210042

**Published:** 2021-05-05

**Authors:** Samantha A. Moore, Angelo Iulianella

**Affiliations:** Department of Medical Neuroscience, Faculty of Medicine, Dalhousie University, and Brain Repair Centre, Life Science Research Institute, 1348 Summer Street, Halifax, Nova Scotia, Canada, B3H4R2

**Keywords:** cortical hem, choroid plexus, hippocampus, neurogenesis, telencephalon, patterning

## Abstract

The dorsal medial region of the developing mammalian telencephalon plays a central role in the patterning of the adjacent brain regions. This review describes the development of this specialized region of the vertebrate brain, called the *cortical hem*, and the formation of the various cells and structures it gives rise to, including the choroid plexus, Cajal–Retzius cells and the hippocampus. We highlight the ontogenic processes that create these different forebrain derivatives from their shared embryonic origin and discuss the key signalling pathways and molecules that influence the patterning of the cortical hem. These include BMP, Wnt, FGF and Shh signalling pathways acting with Homeobox factors to carve the medial telencephalon into district progenitor regions, which in turn give rise to the choroid plexus, dentate gyrus and hippocampus. We then link the formation of the lateral ventricle choroid plexus with embryonic and postnatal neurogenesis in the hippocampus.

## Graphical abstract

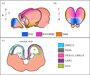

## 1. Patterning the cortical hem

**Figure 1.**
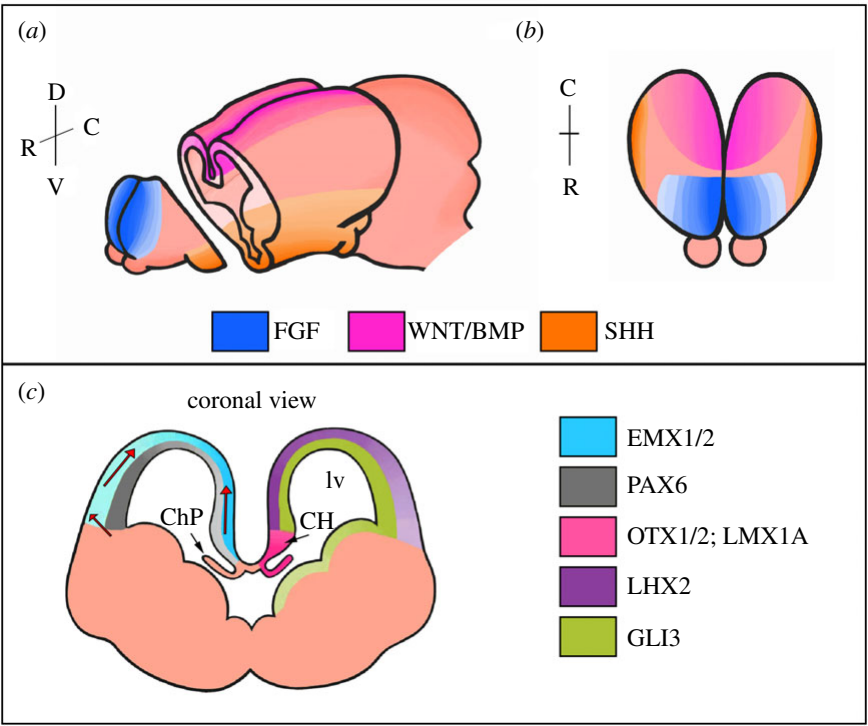
Expression of signalling proteins and transcription factors during forebrain patterning. (*a*,*b*) Expression domains of the principal forebrain morphogens SHH, WNT, BMP and FGF. (*c*) Cross-sectional view of the expression domains of EMX1/2, PAX6, OTX1/2, LMX1A, LHX2 and GLI3 in the developing telencephalon. Red arrows indicate the migration of CR cells. BMP, bone morphogenetic protein; CH, cortical hem; ChP, choroid plexus; FGF, fibroblast growth factor; lv, lateral ventricle; SHH, sonic hedgehog; WNT, wingless–int.

**Figure 2.**
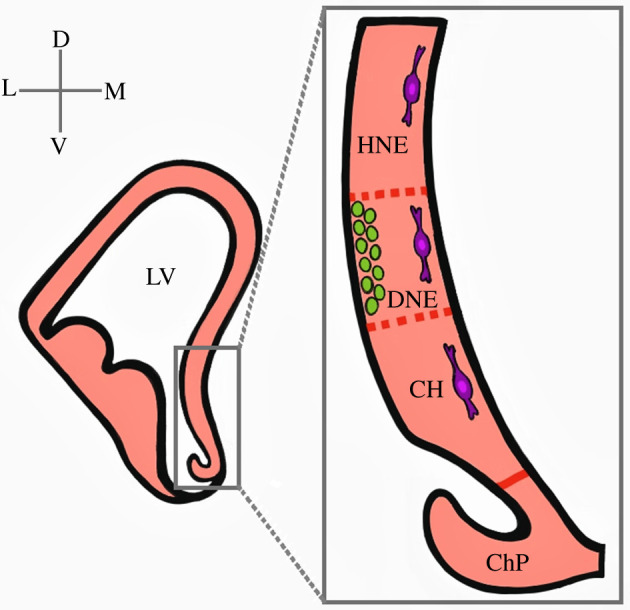
Schematic depiction of fetal cortical hem anatomy. Left side: coronal section at E14.5 displaying the telencephalic midline structures. The CH regulates the induction and formation of hippocampal primordia dorsally and the choroid plexus ventrally. The presumptive DNE is located between the HNE and the CH. Right side: DG precursors (green circles) of the primary matrix are located in the VZ of the ventromedial LV. These precursors migrate to the pial side of the cortex to form the secondary matrix of the DG. CR cells (purple) of CH origin line the pial side of the cortex. CH, cortical hem; ChP, choroid plexus; D, dorsal; DG, dentate gyrus; DNE, dentate neuroepithelium; HNE, hippocampal neuroepithelium; LV, lateral ventricle; L, lateral; M, medial; V, ventral.

The regulation of forebrain patterning has been an area of intense interest over the past few years. One region that serves as a signalling centre that influences the dorsal–medial region of each telencephalic hemisphere is the cortical hem (CH) (figures 1 and 2). At embryonic day 10.5 (E10.5) in mice, the dorsal telencephalic midline (DTM) region folds inward to form complementary telencephalic vesicles, with the resulting midline tissues reorganized to generate the CH [1]. The CH is functionally distinct from the bordering pseudostratified cortical neuroepithelium, playing a crucial role as a classical developmental organizer [2,3]. The CH was initially described as a potential signalling centre in the DTM owing to its enriched expression of prominent morphogen families including wingless/int (Wnt) and bone morphogenetic protein (BMP) [4,5]. By E12.5, the CH is delineated by the expression of three Wnt genes, Wnt2b, Wnt3a and Wnt5a, forming a distinct boundary with the hippocampal primordium dorsally and the CH ventrally [5].

The rostral telencephalic organizer (RTO) has been identified as an additional signalling centre that restricts the boundary of the CH forming region in the medial telencephalon. The RTO is enriched in fibroblast growth factors (FGFs), which control cell behaviour including proliferation, differentiation and migration, and more globally provide positional identity along the rostral–caudal axis of the developing telencephalon (figure 1) [6]. In addition to an inductive role in the telencephalon, the RTO patterns the neuroepithelium into functional domains, defining the anterior limit of the CH [7]. The orchestrated patterning of the DTM region and correct positioning of the CH depend on the antagonistic properties of the RTO source of FGF signalling with Wnt and BMP signalling from the CH. For example, over-activation of BMP signalling represses the transcription factor Lhx2, consequently expanding the CH domain [2]. Conversely, the overexpression of FGFs reduces the CH domain, potentially acting via a BMP-Lhx2 loop to repress the extent of BMP signalling in the medial telencephalon [6–8]. Furthermore, in addition to repressing the extent of *Lhx2* expression in the forebrain, FGF8 signalling decreases *Wnt* expression in the CH domain, thereby defining the anterior boundary of the CH [7]. The proposed feedback pathways add complexity to the balance of signalling morphogens and transcription factors that govern the organization of the telencephalon and ultimately define the boundaries of the DTM.

The role of Wnt signalling in establishing the CH is further supported by the phenotype of the spontaneously occurring mutation, *extra toes-J* (*XtJ*), which affects the *Gli3* gene and results in defects in both neural and limb development. Mice homozygous for the *Gli3 XtJ* mutation lack Wnt and BMP expression within the DTM and lack an identifiable CH [3]. Importantly, the loss of the CH is accompanied by a disruption in the development of the hippocampus and the telencephalic choroid plexus (ChP), with both structures no longer detectable by molecular and morphological markers [4,5]. Specifically, the CH is optimally situated to regulate the induction and appropriate structural organization of the hippocampal primordium dorsally and the ChP ventrally [1,9–11]. Figure 2 depicts the CH region of the embryonic DTM subdivided along the ventral-to-dorsal axis into the ChP, presumptive dentate neuroepithelium (DNE) and presumptive hippocampal neuroepithelium (HNE).

## 2. Origin of Cajal–Retzius cells

During brain formation, a variety of secreted molecules provide the necessary cues for the formation of neural connections. In some regions, guidance cues for axonal pathfinding and target selection are provided by specific cells that exist only transiently during development. Ramon y Cajal [12] and Retzius [13] first described a transient and morphologically complex cell type distributed throughout the marginal zone overlying the fetal and early postnatal mammalian neocortex, known today as Cajal–Retzius (CR) cells. Recent work has centred on the molecular signature and the developmental origin of these cells. The most well-known function of CR cells is their ability to direct neural cell migration and cortical lamination through the expression of the extracellular glycoprotein Reelin [14,15]. However, additional functions have been proposed, including the regulation of the radial glial phenotype [16] and the development of hippocampal connections [17].

CR cells are among the earliest neuronal subtypes to be born in the forebrain. These cells arise from discrete sources within the telencephalon, including the medial CH region of the pallium and the pallial–subpallial boundary [18,19]. The CH forms the principal source of CR cells within the neocortex; these cells co-express Reelin and p73, a transcriptional regulator of the p53-family [20]. CR cells migrate underneath the pia, covering the superficial surface of the cortex and secrete Reelin from the subpallial region [19]. Reelin is a multifunctional protein that plays a critical role in promoting neuronal migration and lamination of the cortex in an inside-out pattern, whereby each cohort of neurons migrate past the established deep layers of earliest-born neurons to form more superficial layers [19,21]. The critical role of Reelin in cortical lamination is further established in the mutant *reeler* mouse where the cortical architecture develops abnormally, with an inversion of cortical layers attributed to a failure of neurons to migrate past earlier-born neurons [19,22,23]. P73 plays an essential role in brain development by regulating the neurogenic pool via promotion of self-renewal and proliferation of immature neural progenitor (NP) cells and functions as a pro-survival factor of mature postmitotic neurons [24].

The complete inactivation of *p73* in mice results in CR cells lacking the expression of Reelin, yet the neocortex develops largely normally in the mutant mice [22,23]. This is due to the fact that residual Reelin-expressing neurons remain in the marginal zone of *p73* mutant mice, which is sufficient to ensure normal development and neuronal survival, and prevent a failure of proper neocortical lamination, which is an anatomical hallmark of the *reeler* phenotype [25–27]. These findings indicate that the production and maintenance of the CR cells depend on p73, but also demonstrate that the mammalian neocortex can tolerate significant losses in CH-derived Reelin-expressing cells.

## 3. Choroid plexus development

The choroid plexus (ChP) of the lateral ventricle is one of the main sources of cerebrospinal fluid (CSF), but its development remains relatively understudied. Only recently has research begun to delve more deeply into the formation of the ChPs and their potential role in neurogenesis [28–30].

### 3.1. Formation and function of the choroid plexus

**Figure 3.**
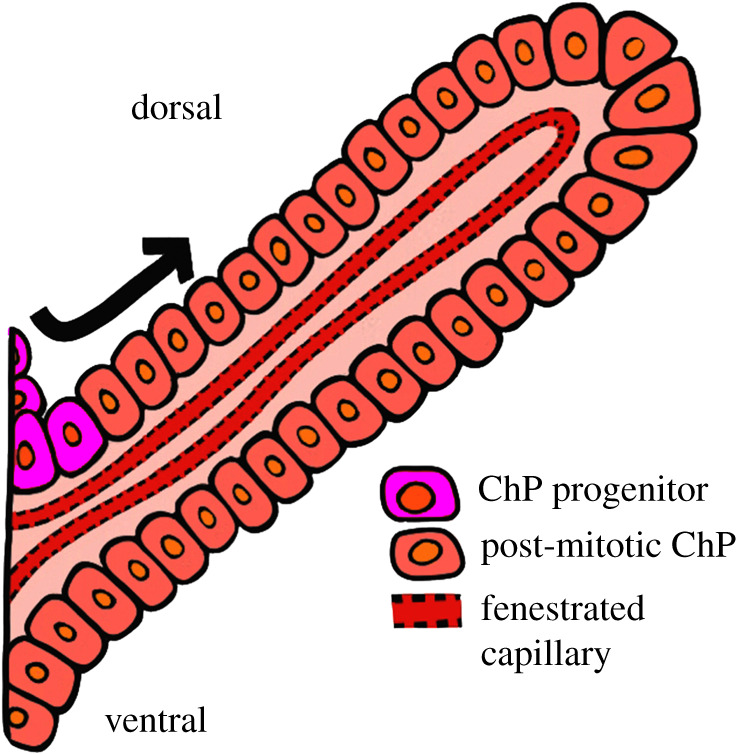
Schematic depiction of choroid plexus anatomy at E14.5. The ChPs are highly vascularized tissue located within each ventricle of the brain. Each ChP consists of a monolayer of cuboidal epithelial cells surrounding a stromal core of capillaries and loose connective tissue. ChP progenitor neuroepithelial cells (violet) originating from the ventricular zone are ‘pushed’ out from the root of the ChP in the upper (dorsal) arm only, by newly divided cells, and migrate towards the tip of the stalk (black arrow indicating direction of cell migration). ChP, choroid plexus.

The ChPs are modified epithelial structures that protrude into all cerebral ventricles (figure 3). They consist of a central stroma that is highly vascularized, with fenestrated, leaky blood vessels and connective tissue [31–33]. The initial development of the ChPs begins around E11.5 and is largely complete by E14.5 in the mouse [34]. The development of these critical components begins with the hindbrain ventricular ChP differentiating first, followed by the differentiation of the lateral ventricular ChPs and finally the differentiation of the third ventricular ChP [29,34]. The ChPs are of dual embryonic origin, with neuroepithelial cells giving rise to the epithelial component, and mesenchymal cells giving rise to the stromal component (figure 3) [31]. The epithelial cells of each ventricular ChP mature through the same stereotypical stages before reaching maturity as functional secretory structures.

In Stage I, the ChP epithelial cells appear pseudostratified with centrally located nuclei. At this early point, there is little to no villous elaboration. Transition to Stage II involves a change to columnar epithelium with apically located nuclei and emerging basal connective tissue with sparse villi-like extensions. This is followed by a transition to Stage III in which the epithelial cells flatten to become more cuboidal in shape defined by centrally or apically located nuclei and more complex villi. By the final stage, Stage IV, the epithelial cells have fully transitioned to a cuboidal morphology, becoming slightly smaller in size and defined by centrally-to-basally located nuclei [32]. Shortly after formation, the ChPs acquire barrier, secretory and transport capacities with cells continuously being added to each ChP throughout early development [31–33]. These epithelial cells are added from the proliferative zone, located at the ‘root’ of each plexus, as they transition through the stages outlined above [29].

The complex molecular relationship between the CH and the developing ChPs has been analysed extensively. Telencephalic ChP formation requires signals from the dorsal midline, including BMPs and Wnts emanating from the CH [5,35–38]. Wnt genes in the CH are upregulated as the ChPs begin to form, supporting a role for CH signalling in the induction of the ChP [5]. Moreover, the mutation of *Gli3*, which can act downstream of Wnt and Shh signalling, results in aberrant CH patterning [5,39,40]. Wnt activity in the CH is important in upregulating the repressor form of Gli3, which in turn suppresses Shh signalling in the anterior–posterior patterning of the developing forebrain neuroepithelium. Shh signalling in turn can feedback to repress Wnt signalling, as was demonstrated during the formation of craniofacial structures and nerves [41,42]. This suggests that the loss of the telencephalic ChPs of the *XtJ* mutant is due to defects in the CH patterning that include the misregulation of Wnt expression [5]. In addition, the CH provides a source of BMPs crucial for the formation of the telencephalic ChPs [36,43]. It is thought that high levels of BMPs are required to induce ChP formation and generation of the thin monolayer of the secretory epithelium [35,38]. BMPs may regulate morphogenesis of the telencephalic ChPs by establishing a balance between restricted cell proliferation and local cell death of the DTM. The dependency of ChP development on CH signalling is further illustrated in mutants affecting the formation of the CH, which also display deficiencies in ChP morphogenesis [1,5,14,44].

Expression of Homeobox transcription factors are also crucial for the development of the CH and ChPs. Opposing gradients of Emx2 and Pax6 expression act cooperatively with Otx1 and Otx2 to ensure the proper development of the caudal forebrain, which includes the CH and adjacent structures (figure 1) [45–47]. Otx2 acts as a master regulator of ChP development [48]. When Johansson *et al.* [48] deleted Otx2 during mouse forebrain development by crossing a tamoxifen-inducible *Otx2^CreERT2^* driver line with *Otx2^fl/fl^*, they found that ChP morphogenesis did not initiate. The severity of the ChP phenotype correlated with the timing of tamoxifen-induced Otx2 deletion, with an early tamoxifen dose resulting in a complete absence of Otx2-immunopositive cells and near-complete loss of all ChP tissue. Therefore, the CH plays a critical role in the initiation and morphogenesis of the ChP, which in turn plays a key role in stimulating neurogenesis from pools of neural stem cells (NSCs) that reside near the ventricles [36,49]. Figure 3 depicts the anatomical organization of the early primary ramus of ChP at fetal stages. Otx2-positive progenitor cells line the dorsal (upper, arrow) root of the plexus and supply postmitotic epithelial cells that fuel the outgrowth of the ChP.

### 3.2. Role of the choroid plexus in the formation of CSF and neurogenesis

NSCs are located at the apical (ventricle facing) surface of the developing brain [50]. During development, NSCs are regulated by several signalling factors enriched within their ventricular and subventricular niches [51–53]. Another potential source of the extrinsic regulation of NSCs is the CSF, which is a fluid secreted from the ChP found throughout the canal structure of the fetal and adult brain and spinal cord, and contains a variety of signalling factors, including those of the TGF-β family [53–55]. The composition of the CSF during development may influence cell behaviour at the ventricular surface, potentially playing a critical role in stimulating neurogenesis. As such, understanding the development of the epithelial and stromal architecture of the ChP is essential to advance our view of the dynamic regulatory mechanisms influencing NSC biology [31,49].

The ChP is an evolutionarily conserved structure that consists of a monolayer of cuboidal cells surrounding connective tissue stroma containing permeable capillaries [28,30–34,36]. The ChP forms early in development from the neuroepithelial cells that line the ventricles and is first detected in mice at E11.5–E12.5 [31]. Initially forming as an outgrowth invading the lumen of the ventricles, ChP epithelial cells are highly organized and joined together by tight junctions, forming the blood–CSF barrier (figure 3). Each ventricular ChP is very active in terms of both protecting and regulating the internal environment of the brain via the blood–CSF barrier, and the secretory function of the CSF begins as soon as the plexuses first appears during development [29]. This timing coincides with the elaboration of forebrain dorsal midline structures, highlighting the importance of the CH in the morphogenesis of the ChP and ultimately in regulating adult neurogenesis from another derivative of the CH, the hippocampus.

Recent studies have shown that the ChPs produce and secrete growth factors that promote neuronal differentiation [49,56]. Shh is an example of a signalling molecule secreted by the ChP during development to promote the expansion NSCs. Its deletion from the mouse hindbrain ChP using the *Wnt1^Cre^* driver resulted in a greater than 50% decrease in the proliferation of NSCs within the surrounding cerebellum [57,58]. In addition, CSF flow mediated by ependymal cells in the subventricular neurogenic niche of the adult rodent brain promotes the migration and guidance of neuronal progenitors [56]. Factors secreted by the ChPs into the CSF bathe the NSC ventricular niche with key regulators of neurogenesis, progenitor survival, proliferation and gliogenesis, stimulating the formation and migration of newborn neurons in the developing and adult mammalian brain [31,59,60]. Despite these recent advances, relatively little focus has been placed on the development of the ChP, and how its aberrant formation may affect CSF secretion and adult neurogenesis. Importantly, the regulation of neurogenesis by molecules produced and secreted from the plexuses could have potentially far-reaching ramifications for the understanding and treatment of neurodevelopmental disorders and pathological neurological conditions associated with ageing [61–63]. While recent findings have led to tremendous progress in our understanding of the role CSF in neural development, future work needs to further elucidate the dynamic morphogenetic processes guiding ChP formation, identify CSF factors that promote neurogenesis and how altered development of these structures contributes to brain disorders.

## 4. Development of the hippocampus

**Figure 4.**
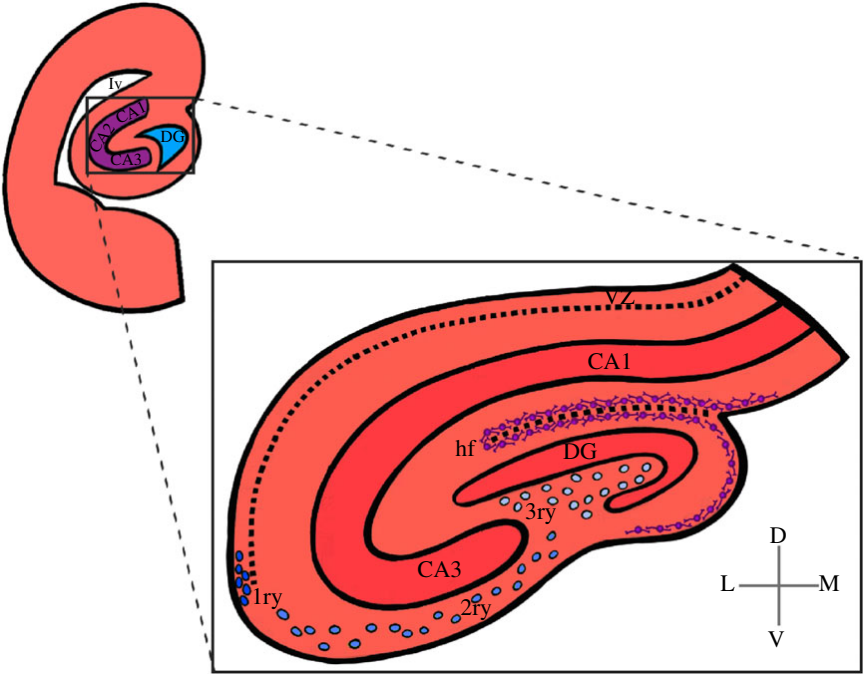
Representation of the mouse hippocampus anatomy at birth. Top left: schematic view of the hippocampus forming region of the cortical hem of the fetal mammalian brain. Bottom right: close-up schematic view of hippocampus anatomy. Granule cell neurons in the DG begin to first appear in the upper blade below the hippocampal fissure. The continuous migration of CR cells reaches the pial side and promotes the formation of the lower blade of the DG. Precursors cells of the primary (dark blue circles) and secondary matrix (blue circles) soon disappear, but cells of the tertiary matrix (light blue circles) continue to actively divide and produce granule neurons throughout postnatal DG development. CA1, cornu Ammonis 1; CA3, cornu Ammonis 3; DG, dentate gyrus; D, dorsal; hf, hippocampal fissure; L, lateral; M, medial; V, ventral; VZ, ventricular zone; 1ry, primary matrix; 2ry, secondary matrix; 3ry, tertiary matrix.

The last major derivative of the CH is the hippocampus, which forms a critical structure within the limbic system and plays an important role in memory formation, especially the transformation of short-term memory to long-term memory [64–68]. Also known as the archicortex, the hippocampus is a phylogenetically ancient region of the brain and is located at the caudomedial edge of the neocortex deep within the medial temporal lobe [69–72]. The hippocampus forms a ‘C’ shape along its longitudinal axis, with the transverse axis divided into distinct fields (figure 4). From proximal to distal, these fields are the dentate gyrus (DG), cornu Ammonis 3 (CA3), and cornu Ammonis 1 (CA1) fields of Ammon's horn, and the cornu Ammonis 2 (CA2) small transitional field. A hippocampal formation is located in the temporal lobe of each cerebral cortex, located along the medial portion of the lateral ventricle's inferior horn. The DG is a separate structure, consisting of granule cells tightly packed in a laminated manner, wrapping around the end of the hippocampus proper [73]. This region of the hippocampus contains NPs and continually supplies newborns neurons postnatally [74–76]. By contrast, the CA areas are filled with densely packed pyramidal cells similar to those found in the neocortex [77–79].

### 4.1. Formation of the hippocampus from the cortical hem

The hippocampus originates from the subventricular region of the dorsal midline of the forebrain in response to signalling from the CH (figure 2). Specifically, the CH region adjacent to the hippocampal primordia expresses Wnts and BMPs, which, as we discussed above, regulate the patterning and growth of the forebrain (figure 1) [1,4,5]. The CH regulates the expansion and development of the caudomedial margin from which the hippocampus develops [1,3,5,44]. Wnt signalling plays an important role in this process. Lee *et al.* [44] found truncated hippocampi in mice lacking *Wnt3a*, the earliest identified Wnt gene selectively expressed in the CH [44]. By contrast, the patterning of the neighbouring neocortex and telencephalic ChP appear to be largely unaffected. In addition, loss-of-function analysis of the transcriptional effectors of Wnt signalling in the forebrain, namely *LEF* and *Gli3*, result in abnormal hippocampus formation and migration of DG precursors [5,44,80–82]. Additional evidence that the CH induces hippocampal fate came from the analysis of *Lhx2* null chimeric embryos [10]. The loss of this transcription factor resulted in the development of multiple hippocampal fields adjacent to each patch of ectopic hem tissue, recapitulating the normal spatial relationships of the CH and hippocampal formation, demonstrating that the CH acts as a hippocampal organizer and is sufficient to induce the specification of hippocampal fields [10,83].

The graded expression of Homeobox transcription factors are also critical for hippocampal development. These include the products of *Emx2*, *Otx1/2* and *Pax6* genes (figure 1). By analysing an allelic series of compound loss-of-function mouse mutants for these genes, Kimura *et al.* [45] discovered that *Emx2*, *Pax6* and *Otx1/2* gene dosage is important for the development of the caudomedial telencephalon, which includes the archicortex (i.e. hippocampus), CH and ChP. Thus, the CH is necessary and sufficient to induce the formation of the hippocampus proper and the migratory progenitors that supply the initial germinal field—the primary matrix—of the DG (figure 2).

### 4.2. Development of the dentate gyrus and germinal matrices of the hippocampus

Development of the DG begins around E12.5 in a complex process involving the migration of the proliferative progenitors away from the DNE (the primary matrix) located between the HNE and the CH [74,84,85] (figure 2). The formation of fated DNE precursors migrate inward away from their sites of origin near the lateral ventricle to generate a subventricular zone (SVZ) of proliferating dentate progenitor cells within the medial telencephalon (figure 2). At E14.5, CH-derived CR cells instruct these precursors to begin migrating toward the pial side of the cortex to form the secondary matrix [85–87]. At this time, radial glial precursors begin to form hippocampal neurons in the HNE. Soon thereafter dentate precursor cells migrate away from the hem region and accumulate at the newly formed hippocampal fissure to form the tertiary matrix or subgranular zone (SGZ; figure 4). CR cells, and a glial scaffold that connects the hippocampal fissure and pial surface, play essential roles in the migration and organization of dentate precursor cells and granule neurons [87,88]. Granule cells generated during DG development come from precursors of all three germinative matrices and contribute to the formation of the dentate blades [74,85,87]. Interestingly, in contrast to the neurogenesis pattern of the pallial neocortex, the developing DG forms in an outside-in manner, with the earliest-born maturing granule cells occupying the outer dentate blade and the more recently generated immature granule cells occupying progressively deeper regions of the blade closer to the hilus [73–75,89]. Precursors in the primary and secondary matrix soon disappear, with subsequent proliferating progenitors being supplied only within the tertiary matrix/SGZ, which is the hippocampal NSC niche contributing to postnatal DG development and neurogenesis [76,85,87] (figure 4). Fate mapping studies from our laboratory and that of others have shown that Cux2 is initially active in the CH, and later becomes restricted to the germinal matrices of the developing hippocampus [74,90]. Specifically, we showed *tdTomato* reporter activity in the *Cux2^ires-Cre^*; *R26r^tdTomato^* mouse line correlates with the outside-in formation of granule cells in the developing and perinatal hippocampal germinal matrices [74]. This confirms the view of the lineage relationship between the medial CH region and DG precursors.

## 5. Conclusion

The CH acts as a signalling centre to promote the diversification of the DTM into distinct progenitor regions that generate the ChP, DG and CR cells. CH patterning is regulated by the Wnt and BMP signalling pathways and several key transcriptional regulators, including the most-anteriorly expressed Homeobox genes—Otx1/2, Emx2 and Lhx2. The Wnt effectors LEF and Gli3 also play key roles in the development of the CH and its derivatives. Our objective here was to give an overview of the dynamic morphogenic processes acting within the CH to diversify the development of forebrain tissues. Furthermore, we highlight that the developmental connection between ChP and hippocampus has consequences for the regulation of neurogenesis in the adult brain through the production of secreted morphogens and chemokines in the CSF.

## Data accessibility

This article has no additional data.
